# Looking to the future of depression: instant diagnosis and medication-free treatment

**DOI:** 10.2144/fsoa-2019-0087

**Published:** 2019-09-10

**Authors:** Daniel Mansson

**Affiliations:** 1 Co-founder & Clinical Psychologist, Flow

## Abstract

Daniel Mansson is a clinical psychologist, cofounder and CEO of Flow. As a clinical psychologist at Sarpsborg DPS, a psychiatric clinic in Norway, Daniel performed extensive investigations and treatments for patients with conditions including depression and attention deficit hyperactivity disorder. In 2012, Daniel met Erik Rehn, where they both worked in the research department of computational biology at KTH Royal Institute of Technology, Stockholm. In January 2016, with combined backgrounds in clinical psychology, computer networks, computational neuroscience and electrical engineering, they launched Flow. The mission was simple: to find new ways to treat mental health issues with the help of science and technology. Based in Malmo, Sweden, Daniel is the driving force behind Flow and spearheads the research, innovation and business development.

## Please tell us about your background & how you got to where you are today

My professional background comprises two fields; IT and psychology. My career started with a BSc in Computer Networks, and as a specialist building and running networks and large IT infrastructures. Several years later, I decided to train as a psychologist. Graduating in 2014, I practiced as a clinical Psychologist at a psychiatric clinic in Norway, diagnosing and treating patients with various mental health conditions including depression, anxiety, severe trauma, OCD, personality disorders and schizophrenia.

During my psychology studies, I read more about a type of treatment where weak electrical currents are sent into the brain to activate neural activity. This technology is called Transcranial Direct Current Stimulation (tDCS). I followed this field closely. In fact, I focused my Masters thesis on tDCS as a method for positively affecting brain activity. Work from my thesis was later published in a peer-reviewed scientific journal.

Soon afterward, I took part in scientific conferences worldwide and discussed tDCS breakthroughs with some of the most respected researchers in the field. It soon became apparent that we were at an important point, scientifically, were we where able to use this method for the treatment of depression. tDCS was proven safe and efficient to treat patients at home, without the many side effects that usually accompany a medication approach. Greater access to this treatment meant that patients across the EU could now use an effective breakthrough for the treatment of their mental health and, specifically, depression in a couple of days.

## You co-founded Flow: can you tell us more about Flow, & what led you to start it up?

Flow is a medical device company with a mission to use technology, psychology and neuroscience to create new treatments for mental health conditions. Technology helps to reach patients in a quick, efficient way. It also helps to improve the product in a more powerful, productive way than if we were to develop a new drug as we can see how people react to the treatment and improve as we go.

We believe treatment is most effective when people are offered this technology together with knowledge as to how they can change their mood. Our first product, Flow, combines a brain stimulation headset and virtual therapy app, which helps users to change their behaviour in four areas; sleep, exercise, nutrition and meditation – each of which are supported by a large body of clinical evidence. This integrated approach, technological and psychological lifestyle change, is the foundation of Flow.

## Flow has been approved in Europe as a medication-free treatment for depression. What are the problems with the current state-of-the-art for antidepressants?

Antidepressant medication has helped many people live their lives without depression. More people should be encouraged to seek help for their depression – and consider all the options available, including antidepressants.

The problems that many people experience with antidepressants are the side effects. Typically, this means people stop using them – or the antidepressants cause new issues including sexual problems, weight gain or heightened anxiety. This poses a real problem for patients.

## How does it work?

Flow consists of a brain stimulation headset and a therapy app which are used together as both have roles to play. The headset sends a gentle electrical signal. This excites neurons on the left frontal part of the head which rebalances neural activity in this area. And this is important as it has been shown that, in general, people with depression have less neural activity in the left frontal part of the brain.

During brain stimulation, the app teaches users what depression is and what can be done about it by changing lifestyle behavior in four areas: sleep, exercise, nutrition and meditation.

The headset is controlled by the app and is used for 30 min/day over a 6-week period. Treatment can continue one- to two-times per week after the 6-week period is over.

## How has it fared in trials (please give a brief overview of results)?

tDCS has been studied since the early 1960s with an increasing amount of independent randomized controlled trials conducted over the last decades. There are 500+ articles published, each examining the effectiveness of tDCS and other electric brain stimulation techniques.

The area that we are most interested in is tDCS and depression. There are 10+ positive randomized controlled trials published in respected scientific journals, including the *New England Journal of Medicine* and *British Journal of Psychiatry*.

In a recent study published in *New England Journal of Medicine*, a double-blind placebo-controlled study showed that 41% of patients using tDCS experienced a 50% reduction or more in their depression. The effect of the antidepressant used in the study (escitalopram) was slightly higher than tDCS (47% of people experienced a 50% reduction in depression) [1].

## What's next?

This summer, Flow launched in the UK and Sweden. We will continue to improve the app and are opening up the treatment in other EU countries. Our collaboration with the scientific community is going from strength to strength. During late 2019, we are conducting a randomized controlled trial with professor Andre Brunoni (University of Sao Paolo, Brazil), one of the leading researchers in the field of tDCS and depression, and we have recently secured $1.5 million investment from Khosla Ventures for funding our European rollout and further clinical studies. We are also planning to launch Flow in several psychotherapy clinics in the UK. Our intention is that Flow will complement other techniques (such as talk therapy) used by psychotherapists in these clinics.

## What's your dream for the future of treatment for depression?

Typically, people suffering with depression, particularly in the UK, have a long time to wait until treatment commences.

At Flow, we want to create a world where people have instant access to a diagnosis and subsequent treatment for their depression, without side effects. The WHO estimates that over 300 million people have depression, and have named the condition as the world's leading cause of disability. Flow, and other companies in this field, are continuing to advance technology to make tDCS treatment more efficient. We believe the path forward is personalization and this should be pursued with a great sense of urgency to ensure everyone can get access to treatment.
